# Similarities Between Depression and Neurodegenerative Diseases: Pathophysiology, Challenges in Diagnosis and Treatment Options

**DOI:** 10.7759/cureus.11613

**Published:** 2020-11-21

**Authors:** Madiha Hussain, Prabhat Kumar, Sara Khan, Domonick K Gordon, Safeera Khan

**Affiliations:** 1 Neuropsychiatry, California Institute of Behavioral Neurosciences & Psychology, Fairfield, USA; 2 Medicine and Surgery, Bangalore Medical College & Research Institute, Bangalore, IND; 3 Gastroenterology and Hepatology, Cleveland Clinic Foundation, Cleveland, USA; 4 Internal Medicine, California Institute of Behavioral Neurosciences & Psychology, Fairfield, USA; 5 Internal Medicine, Scarborough General Hospital, Scarborough, TTO

**Keywords:** depression pathology, neurodegenerative diseases and depression, neuroinflammation, stress and depression, treatment of depression in alzheimer's disease, treatment of depression in parkinson's disease

## Abstract

Depressive disorder and neurodegenerative diseases are two different clinical entities. Depression is a common psychiatric disorder in the general population. However, when present concomitantly with neurodegenerative disorders, its diagnosis becomes challenging. In many cases, patients remain undiagnosed and hence, untreated, worsening the prognosis of the neurodegenerative diseases and impairing the quality of life.

One of the possible reasons for the difficulties in diagnosis in such cases is that both conditions affect the central nervous system, so there might be an overlap of symptoms leading to a missed diagnosis of depression in a neurodegenerative disease patient and vice versa. Symptoms such as irritability, apathy, and decreased cognition are common to both types of disorders. Some neurodegenerative diseases, especially Alzheimer's disease, can initially present as a depressive prodrome. This may cause a difficulty in differentiating between these two conditions and a diagnosis of either conditions may be missed; hence an opportunity for timely intervention and improved outcomes is missed.

An approach towards analyzing and comparing the pathological mechanisms common to both disease types will create a better understanding of depression and neurodegenerative diseases, identify their similarities, and develop improved clinical criteria to help clinicians make a timely diagnosis of these conditions present together. In the present review, various studies related to common pathological links, concomitant diagnosis challenges, and ongoing research about different treatment options are discussed.

## Introduction and background

Depression is a common psychiatric disorder and contributes significantly to the disease burden on healthcare systems worldwide. According to one study, about 322 million people, about 4.4% of the global population, suffer from depression at any given time [1]. It negatively affects the overall quality of life along with normal day to day functions. Clinical depression leads to an increased risk of worse prognosis among patients with chronic diseases [2]. Depression is among the most prevalent psychiatric disorders in the elderly population [3].

The incidence of neurodegenerative diseases increases with increasing age and is frequently seen in the elderly population. Some common examples of neurodegenerative diseases include Alzheimer's disease, Parkinson's disease, and Huntington's disease. Several pathological factors contribute to the neurodegenerative process, including genetic predisposition, environmental factors, and the normal aging process along with many others.

Depression and neurodegenerative diseases are clinically recognized as two entirely different entities; however, their concomitant presentation has been increasingly identified. The depression rate can be as high as 90% in Alzheimer's disease and 50% in patients with Parkinson's disease [2]. This common co-occurrence of these disorders raises many questions regarding the pathophysiological links between these disorders. For example, can depression predispose a patient to neurodegenerative diseases? Does neurodegeneration play a role in the development of major depressive disorder in the elderly? Is there any pathological explanation for this frequent co-occurrence of these diseases? Although no clear mechanism has been identified yet, several mechanisms that co-exist in depression and neurodegenerative diseases have been implicated.

One of the most widely accepted mechanisms is neuroinflammation. It is seen in both depression and neurodegenerative diseases. Several studies show that pro-inflammatory cytokines are raised in the cerebrospinal fluid (CSF) of patients with depression and those with neurodegenerative diseases [4]. This makes neuroinflammation at least partially responsible for the association between the two disorders. 

Another widely studied concept is the monoamine oxidase pathway. Several monoamine neurotransmitters, for example, serotonin, dopamine, and nor-epinephrine, have been implicated in the pathophysiology of many neurodegenerative diseases, like dopamine in Parkinson's disease, glutamate in Alzheimer's disease, etc. It is also a well-established fact that monoamines, especially serotonin, play an important role in depressive disease. Several monoamine oxidase inhibitors (the enzyme responsible for the degradation of monoamines) are used to treat depression, Parkinson's disease, and Alzheimer's disease [5].

Other associations like hypothalamus-pituitary-axis dysfunction are seen in both depression and neurodegenerative disorders, clinically assessed by a decrease in the CSF somatostatin-like immunoreactivity (SLI) [4]. Also, decreased levels of brain-derived neurotrophic factor (BDNF), which plays an important role in neuroplasticity, and increased oxidative stress levels have been associated with both conditions [4].

Clinically, this common co-occurrence of depression and neurodegenerative diseases poses two types of challenges. The first main challenge is the differential diagnosis because neurodegenerative diseases have several psychiatric manifestations, including cognitive decline, apathy, and irritability, which are also seen in depression [6]. In some cases, particularly in Alzheimer's disease, the initial presentation is a depressive prodrome [7]. Therefore, the diagnosis of Alzheimer's disease can be missed without careful clinical evaluation. Similarly, depressive symptoms may be overlooked in patients with neurodegenerative diseases in favor of these diseases' psychiatric manifestations. This dilemma can lead to suboptimal treatment and a worsened prognosis.

Another challenge is the treatment of depression in the presence of neurodegenerative diseases. Studies show that even after the diagnosis of depression in the presence of neurodegenerative disease, it is resistant to traditional anti-depressant therapy. The resistance may be partial or complete, leading to a further decrease in cognition and quality of life [8].

Despite a common association between depression and neurodegenerative diseases, there is not enough research that identifies a clear pathological correlation, which has given rise to difficulties in treating the two conditions when they present together. Also, there is a need to develop clinical criteria to differentiate and diagnose these conditions when they co-exist. 

Depression, when present alone in an individual, causes a significant decline in cognition, affects daily functioning, and impairs quality of life. A concomitant presentation of depression and neurodegenerative disease further decreases the cognition and function of an already degenerating brain. Depressed patients with neurodegenerative diseases have a worse clinical prognosis than non-depressed patients with neurodegenerative diseases [4]. Although depression itself is not considered a neurodegenerative disease, its high prevalence in these diseases and involvement in various common pathophysiological pathways with these diseases suggests that not only is there a lack of research on the establishment of the causal relationship between these conditions, but also depression and neurodegenerative diseases as separate entities are still poorly understood. This article will analyze different pathological correlations between the two conditions and address the difficulties in diagnosis and various treatment options.

## Review

Methods

We conducted a literature search online via Google Scholar, PubMed/Medline, Cochrane, and Embase, utilizing eight main combination keywords, as listed in Table 1.

**Table 1:** Electronic Search Results after Using Each Keyword Combination.

Keywords used	Databases Searched	Number of Results	Date
Depression and Neurodegenerative disorders	PubMed Google Scholar	12,974 210,000	09/08/2020
Neuroinflammation and Depression	PubMed Google Scholar	1,254 82,100	09/08/2020
Neuroinflammation and Neurodegenerative Diseases	PubMed Google Scholar	5,846 140,000	09/08/2020
Stress, Depression and Neurodegenerative Diseases	PubMed Google Scholar	1,099 118,000	09/08/2020
Treatment of Depression in Neurodegenerative Diseases	PubMed Google Scholar	6,752 29,900	09/08/2020
Similarities between Depression and Neurodegenerative Diseases	PubMed Google Scholar	950 41,700	09/08/2020
Depression, Neurodegenerative Diseases and Anti-depressant Therapy	PubMed Google Scholar	1,063 36,800	09/08/2020

Various abstracts were selected based on the study's topic, and after careful evaluation of each abstract, each study's relevance was determined. The search results showed various types of studies; the majority were review articles and some clinical trials. We gained access to the full articles of relevant studies via online purchases, librarians, and some were available free online in the search engines. Some relevant studies were also found in the reference lists of the selected studies. We finalized around 30 full articles to use as our literature to be reviewed.

Selection Criteria

We included all study types and geographical locations. We only included articles published in the English language, and all the studies included were published in the last 20 years.

Results

Among the studies we selected, six were observational studies, three were systematic reviews and meta-analysis, two were randomized controlled trials (RCTs) and the rest of the studies were review articles. The collected articles discussed various pathologies common to both depression and neurodegeneration. In this article a bigger picture of how these mechanisms come together and play a role in developing these disorders is painted. Then the clinical aspect of diagnostic challenges and their implications is discussed along with resistance to treatment, and new avenues of research for improved therapeutic outcomes are outlined.

Discussion

Pathophysiological Pathways Common to Both Depression and Neurodegenerative Diseases

As mentioned above, it is unclear how exactly and to what extent these mechanisms contribute to predisposing the patients of neurodegenerative diseases to depression and vice versa. Several factors contribute to the decline in cognition and other common symptoms of both these diseases.

Neuroinflammation

Inflammation in the central nervous system has been implicated in developing various neuropsychiatric disorders, including depression and neurodegenerative diseases. It is one of the most intensely researched topics in neuropsychiatry. Although the monoamine oxidase theory is the most widely known in the depressive disease's pathophysiology, various other mechanisms have been proposed to contribute to its pathophysiology in recent years. Neuroinflammation has gained popularity in this regard in recent years. The same is true for neurodegenerative diseases. Several pathways revolving around inflammation are thought to be involved, but the exact mechanisms are not fully understood.

The central nervous system's microglial cells are the key players in regulating the brain's immune response. These cells also contribute to the processes of neuronal degeneration and neurogenesis [9,10]. Interestingly, these cells are seen to be more active in patients of depression, as well as neurodegenerative diseases [11-13]. Under normal circumstances, these cells maintain a balance between pro-inflammatory and anti-inflammatory processes in the central nervous system (CNS). However, studies show that prolonged activation of these cells tips the balance in favor of pro-inflammatory processes leading to increased pro-inflammatory cytokines such as tumor necrosis factor-alpha, interleukin (IL)-1 beta, and interleukin-6. Anti-inflammatory cytokines such as IL-10 and neurotrophic factors such as BDNF are decreased [11].

Many studies show that the levels of pro-inflammatory cytokines, e.g., interleukin-6, are raised in the cerebrospinal fluid of patients with neurodegenerative diseases and patients with depression [11]. Interestingly the demonstration of neuropsychiatric symptoms of dementia, like irritability and depressed affect, is more common in patients with higher levels of IL-6 in their cerebrospinal fluid [4]. This further supports the theory that neuroinflammation is a common pathological link between depression and neurodegenerative diseases.

**Figure 1:**
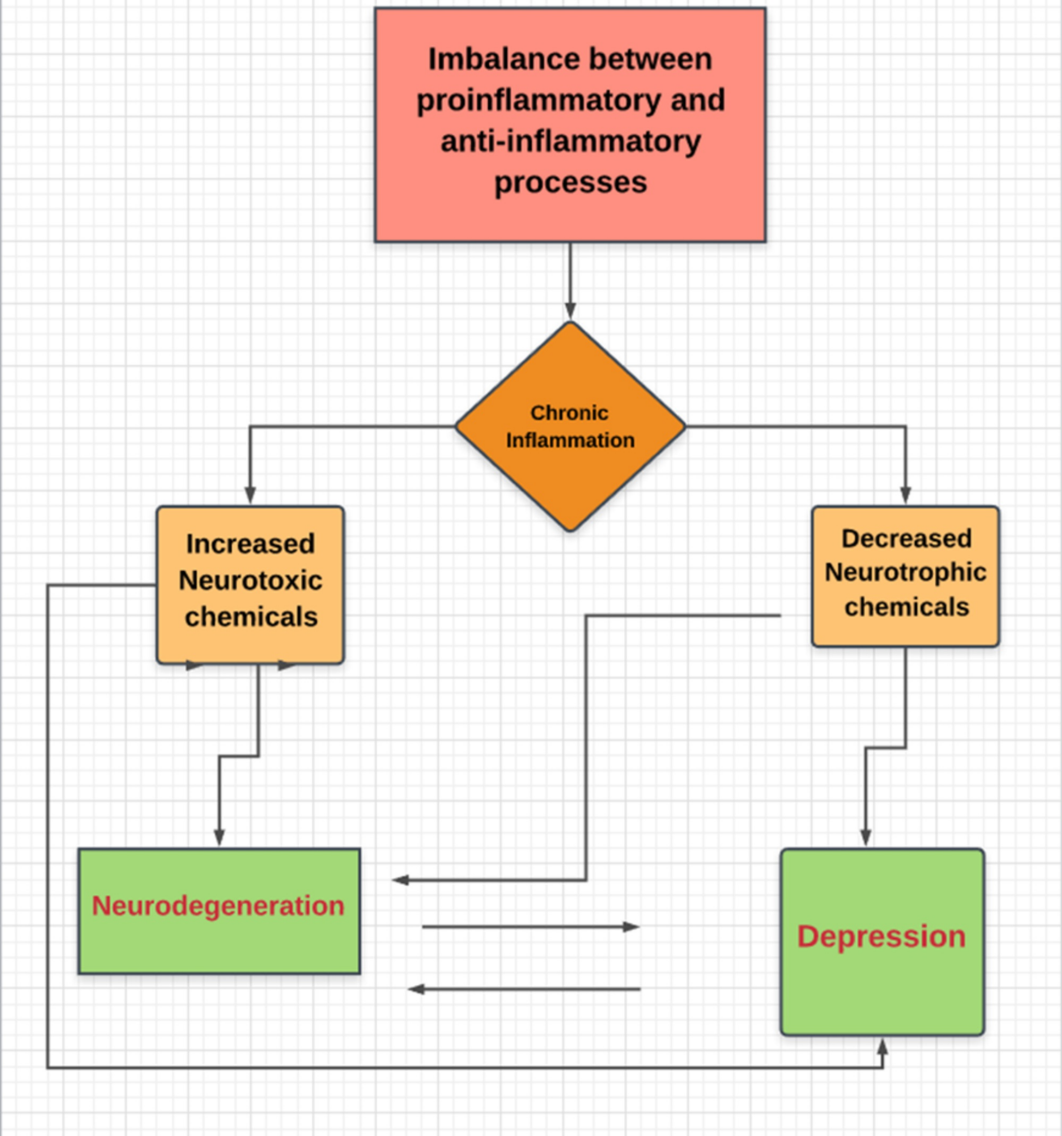
Vicious cycle of neuroinflammation and neuronal injury: Chronic neuroinflammation affects brain via two main mechanisms i.e; decrease in neurotrophic factors and increase in neurotoxin levels resulting in neuronal injury and degeneration which further increases neurotoxins and a vicious cycle ensues.

Acute inflammation in response to an external or internal insult is protective. However, when the pathways responsible for the termination of the inflammatory process and tissue repair initiation fail to function, this results in chronic inflammation. Chronic inflammation leads to the release of neurotoxic substances, increased neuronal degeneration, and decreased neuroplasticity. Figure 1 shows the mechanism of neuroinflammation in neurodegeneration and depression [9].

On the other hand, patients with Alzheimer's disease with lower depression scores and less agitation were shown to have increased levels of interleukin-10, which is an anti-inflammatory cytokine. In summary, increased pro-inflammatory marker levels are associated with higher rates of depression. In comparison, low pro-inflammatory marker levels and high anti-inflammatory marker levels are associated with lower depression rates in patients with a history of neurodegenerative diseases [4].

Monoamine Oxidase Pathways

Alterations in the levels of monoamine neurotransmitters have been implicated in the etiology of several psychiatric and neurological disorders. As far as major depression is concerned, decreased serotonin levels, leading to depressive symptoms, is the most widely accepted theory. Similarly, Parkinson's disease is associated with reduced levels of dopamine. 

Accordingly, the therapeutic regimens for major depression and Parkinson's disease include monoamine oxidase enzyme inhibitors, leading to increased neurotransmitter levels. This results in decreased symptoms of depression and Parkinson's disease.

Moussa B H Youdim, a pioneer in the research for the development of monoamine oxidase inhibiting drugs, published a study in 2006 regarding selective monoamine oxidase inhibitors in treating depression in neurodegenerative disease patients like Alzheimer's and Parkinson's disease [5]. This study gave rise to the idea of neuroprotection through the use of multi-target regimens, which show the potential for not only halting the process of neurodegeneration but also reversing it. The study shows that many complex interactions between free iron levels and monoamine oxidase enzymes in the central nervous system culminate in a neurotoxic environment due to increased oxidative stress levels in the brain, speeding up the process of neuronal degeneration [5].

This study changed the course of pharmaceutical research on depression and neurodegenerative diseases. It brought to light the role of monoamine oxidase enzymes in the pathologies of both types of diseases, making these enzymes a common link between depression and neurodegenerative diseases [5]. In recent years, many new studies have confirmed that monoamine oxidase enzymes play an important role in developing both depression and neurodegenerative diseases [14].

Stress and Hypothalamic-Pituitary-Adrenal Axis Dysfunction

Chronic stress can lead to Hypothalamic-Pituitary-Adrenal (HPA) axis dysfunction. Aging is also a risk factor for HPA axis dysfunction. This dysfunction plays a role in the development of psychiatric as well as neurodegenerative disorders. Like all the other mechanisms mentioned above, this is also involved via complex pathways in developing these disorders. Still, in summary, it causes decreased negative feedback inhibition in the HPA axis, resulting in increased levels of corticotropin-releasing hormone (CRH), adrenocorticotropic hormone (ACTH), and cortisol that leads to neuronal degeneration and depressive symptoms [15]. 

Increased levels of cortisol in response to stress is a normal physiological event that is compensatory. However, suppose the levels of cortisol remain high over extended periods. In that case, this can result in a cascade of events leading to the atrophy of neuronal circuits and degeneration of neurons [15]. The psychiatric manifestations of hypercortisolism are also well documented, especially depression. HPA axis dysfunction and subsequent hypercortisolemia have been associated with memory impairment and atrophy of the hippocampus [15], further supporting the theory that they can result in neuronal degeneration.

Another mechanism through which this system participates in these diseases' pathophysiology is by causing maladaptive changes in the neuronal networks leading to their re-organization [15], which leads to various behavioral and mood disturbances and eventual degeneration of those neurons.

All of these processes culminate in the development of a neurotoxic environment in which neurotrophic factors, e.g., BDNF, production is decreased [4,11]. Hence, not only is the brain subjected to stressful stimuli and toxic insults, its ability to heal itself, and rewire the neuronal circuits, i.e., neuroplasticity, is also compromised. This causes a vicious cycle of toxic injury and stress, which further causes increased neuronal degeneration and depressive symptoms leading to increased stress and so on.

Therefore, the complex interactions between neuroinflammation, monoamine pathways, and chronic stress, along with many other factors, resulted in the overproduction of neurotoxins and decreased production of neuroprotectants, as well as increased oxidative stress, predisposing an individual to be neurologic and psychiatric disorders. This complex interface between neurodegenerative and neuropsychiatric disorders poses more questions than answers.

Although these theories explain an important aspect of this complex pathophysiology, the literature clearly lacks on how all these pathways come together and interact to produce such complex neurodegenerative and psychiatric manifestations.

Challenges in Diagnosis

Neurodegenerative disorders, like any other brain pathology, can have psychiatric manifestations involving mood and behavior. So, suppose a psychiatric disorder, especially depression, is present with Alzheimer's disease or any other neurodegenerative disease. In that case, it can be challenging to diagnose because of the overlap of symptoms between depression and these disorders as depression can also present with neurological symptoms such as cognitive decline [16].

The depressive prodrome is one of the earliest manifestations of neurodegenerative diseases, especially Alzheimer's disease [17,18] as well as Parkinson's disease [8]. In such cases, the patient might only be diagnosed with depression and treated inappropriately, and earlier diagnosis and intervention of a neurodegenerative disease can be missed [17]. 

However, in Alzheimer's disease and other neurodegenerative diseases, depression can present at any stage of disease evolution [18]. Regardless of the time of presentation, most patients suffering from a neurodegenerative disease suffer from depression at some point during their disease. 

According to one study, irritability is one of the most common psychiatric symptoms in dementia patients in nursing homes [19]. Apathy is also reported among these patients [6,20]. These symptoms are commonly overlooked, but they might be signs of underlying depression [6,19,20]. 

These neuropsychiatric symptoms increase disease burden and negatively affect the disease prognosis in patients with neurodegenerative diseases. The quality of life of neurodegenerative disease patients with depression is worse than those of neurodegenerative disease without depression [10,21].

There is a lot of confusion in the literature, and an ongoing debate about whether depressive illness predisposes to neurodegenerative diseases or the presence of such disease in itself is a risk factor for the development of depression if the patient is aware and able to understand the consequences of their illness [20,22].

However, a brain that suffers from depression at an earlier age can undergo significant maladaptive structural and functional changes that may predispose it to the process of neuronal degeneration [18,23]. Some studies propose that pseudodementia of depressive illness and depression in neurodegenerative disease are two ends of a spectrum based on Janet's concept of psychological tension [24]. This method of viewing these conditions via psychological tension theory might help the clinician better understand these disorders; however, there is a lack of substantial research in this area. 

Unfortunately, there are no diagnostic tests or biological markers for diagnosing depression [3]. Clinicians have to rely on patient history and various diagnostic scales and criteria for its diagnosis when present alone. When it presents with other neurological conditions, these tools prove insufficient to rely upon. Hence there is a need to develop biological markers along with better and more concise clinical criteria and a high index of suspicion for depression in the elderly population presenting with neurodegenerative illness [17]. More research needs to be done to develop biomarkers for the diagnosis of depression [3] as well so that timely diagnosis and early intervention can be made to slow down the progression of these conditions, which will result in improved quality of life and better clinical outcomes for patients. 

Treatment Options

Studies have shown that the depression that presents comorbidly with neurodegenerative diseases like Alzheimer's disease and Parkinson's disease is either partially or entirely unresponsive to the traditional anti-depressant treatments [8,10]. Currently, the traditional anti-depressants like selective serotonin reuptake inhibitors and serotonin-norepinephrine reuptake inhibitors in combination with other drugs like cholinesterase inhibitors, monoamine oxidase inhibitors depending upon the type of neurodegenerative disease present along with depression, are being used for the treatment of depression in patients with neurodegenerative diseases [8,10,25].

N-methyl-D-aspartate (NMDA) receptor antagonists like memantine and ketamine are intensively being studied to treat various psychiatric and neurological diseases. Memantine is currently approved by the FDA to treat Alzheimer's disease because of its safety profile and efficacy [26]. Interestingly, NMDA receptor antagonist drugs are also being studied for their anti-depressant properties; especially ketamine has shown remarkable anti-depressant effects in animal studies [27]. Some studies also suggest that these drugs have neuroprotective properties [28], making them stronger candidates for the treatment of depression present with a neurodegenerative disease in the future.

As mentioned previously, selective monoamine oxidase inhibitors are also currently being studied for their use in depression and neurodegenerative diseases [5]. Lodastigil, a reversible acetylcholinesterase and butyrylcholinesterase inhibitor as well as an irreversible Monoamine Oxidase Type B (MAO-B) inhibitor, is currently in phase II of clinical trials for its use as an anti-depressant and neuroprotectant agent for the treatment of depression and various neurodegenerative diseases, including Alzheimer's disease and Parkinson's disease [28]. Hence it is reasonable to conclude that this drug, once approved, will provide better clinical outcomes for patients suffering from both depression and neurodegenerative diseases simultaneously.

Many anti-inflammatory substances like curcumin, alcohol, and resveratrol have shown beneficial effects for both depressive and neurodegenerative symptoms in animal studies [11]. However, the number of studies available is insufficient to ascertain the potential benefits of using anti-inflammatory agents to treat depression and neurodegenerative diseases. Further research in such therapies needs to be carried out to better these patients' quality of life.

Antioxidants' role needs to be further explored, as oxidative stress contributes to neuroinflammation [29]. As a result of HPA axis dysfunction, hypercortisolism is also a possible therapeutic target [30]. Corticosteroid antagonist therapies should also be explored for the treatment of both types of disorders.

## Conclusions

Depression is a prevalent and largely missed diagnosis in neurodegenerative disease patients. It has a significant impact on the prognosis and clinical outcomes as well as the quality of life. However, the neuropsychiatric literature is still not sufficient on this subject to help clearly understand the complex neuropathology of these conditions alone and how they are intertwined to explain this common co-occurrence. 

The concepts of neuroinflammation, neuroplasticity, HPA axis dysfunction, and, more recently, the monoamine oxidase pathways have helped improve the understanding of these diseases immensely. However, more research needs to be done to establish the reason for a very high rate of co-existence of these two conditions, and whether depression is a risk factor or a consequence of neurodegenerative disorders. 

Also, clinically these disorders are confused and missed due to overlapping symptomatology. So, clinicians need to have a high index of suspicion and better clinical evaluation criteria to correctly diagnose depression in the presence of neurodegenerative disease and treat it in a timely manner. Better treatment regimens need to be developed, and it's an area of potential for intensive future research, especially the concept of neuroprotectants and anti-inflammatory regimens.
